# Immune targeting of three independent suppressive pathways (TIGIT, PD-L1, TGFβ) provides significant antitumor efficacy in immune checkpoint resistant models

**DOI:** 10.1080/2162402X.2022.2124666

**Published:** 2022-10-01

**Authors:** S. Elizabeth Franks, Kellsye P. Fabian, Ginette Santiago-Sánchez, Benjamin Wolfson, James W. Hodge

**Affiliations:** Laboratory of Tumor Immunology and Biology, Center for Cancer Research, National Cancer Institute, National Institutes of Health, Bethesda, MD, USA

**Keywords:** TIGIT, bintrafusp alfa, M7824, immunotherapy, combination immunotherapy

## Abstract

Immune checkpoint blockade (ICB) therapy, while groundbreaking, must be improved to promote enhanced durable responses and to prevent the development of treatment-refractory disease. Cancer therapies that engage, enable, and expand the antitumor immune response will likely require rationally designed combination strategies. Targeting multiple immunosuppressive pathways simultaneously may provide additional therapeutic benefit over singular targeting. We therefore hypothesized that the use of two molecules which inhibit three independent, but overlapping, pathways (TIGIT:CD155, PD-1/PD-L1, and TGFβ) would provide significant antitumor efficacy in the syngeneic ICB resistant colorectal tumor model MC38 expressing human carcinoembryonic antigen (CEA) in CEA transgenic mice. This novel combination treatment strategy has significant antitumor activity and survival benefit in two models of murine carcinomas, MC38-CEA (CRC) and TC1 (HPV^+^ lung carcinoma). MC38-CEA mice that responded to αTIGIT and bintrafusp alfa combination therapy generated memory responses and were protected from rechallenge. These effects were dependent on CD4^+^ and CD8^+^ T cells, as well as increased immune infiltration into the TME. This combination induced production of tumor-specific CD8^+^ T cells, and an increase in activation and cytotoxicity resulting in an overall activated immune landscape in the tumor. Data presented herein demonstrate the αTIGIT and bintrafusp alfa combination has efficacy across multiple tumor models, including the checkpoint-resistant model of murine colon carcinoma, MC38-CEA and the HPV^+^ model TC-1.

## Introduction

Over the last several decades, multiple barriers to successful antitumor immunity have been identified, including signals that drive an activating versus suppressive immune response.^1,2^ Upon engagement of immune cells, parallel costimulatory and coinhibitory programs are activated to finely tune the response. Following identification of the first checkpoint molecule, CTLA-4,^3^ multiple additional crucial regulators of the T cell immune response have been identified, including but not limited to programmed cell death-1/programmed cell death ligand 1 (PD-1/PD-L1), TIGIT, TIM3, LAG3, and VISTA. There are multiple clinical trials investigating immune checkpoint blockade (ICB) agents across a myriad of indications^4^ (clinicaltrials.gov). Despite success, albeit in limited capacity, there lies a significant unmet clinical need to treat patients suffering from ICB-refractory malignancies. Here, we strategically designed a combination treatment regimen targeting multiple immunosuppressive pathways using two novel molecules, the fusion protein bintrafusp alfa and a monoclonal antibody targeting TIGIT (αTIGIT).

Bintrafusp alfa is a bifunctional fusion protein composed of the extracellular domain of the human transforming growth factor β receptor II (TGFβRII or TGFβ ”trap”) fused via a flexible linker to the C-terminus of each heavy chain of an IgG1 antibody blocking anti-PD-L1.^5,6^ This molecule has been shown to sequester all three isoforms of TGFβ, and this trap function is physically linked to PD-L1 blockade in the tumor microenvironment (TME).^7^ In the EMT-6 breast and MC38 colorectal murine cancer models, bintrafusp alfa treatment resulted in superior tumor growth suppression and prolonged survival than treatment with anti-PD-L1 or TGF-β trap alone.^7^ Furthermore, there have been several positive responses using bintrafusp alfa in patients with heavily pretreated advanced solid tumors.^6,8,9^

TIGIT is an inhibitory receptor with expression restricted to T cells and natural killer (NK) cells, with highest expression found on regulatory T cells (Tregs).^10^ CD155 is the dominant cognate receptor that interacts with the immunosuppressive receptor TIGIT, and the immunoactivating receptor CD226.^11^ CD226 is to CD28 as TIGIT is to CTLA-4, with TIGIT binding to CD155 in an inhibitory fashion, at much greater affinity (1–3 nM), in comparison to the positive signaling moiety CD226 (115 nM).^12,13^ TIGIT, while constitutively expressed on Tregs, is found in low abundance on naïve cells and is significantly upregulated following antigenic stimulation of T cells. TIGIT can deliver inhibitory signals in a paracrine and autocrine fashion, through binding with CD155 and direct disruption of homodimerization of CD226, respectively.^14^

TIGIT can interfere with the cancer immunity cycle in a variety of ways, including suppression of NK^12^ and  CD8^+^ T cell-mediated killing,^11^ induction of immunosuppressive dendritic cells (DCs),^10^ and skewing CD4^+^ and CD8^+^ T cell priming and differentiation to immunosuppressive phenotypes.^15,16^ Data pooled from the TCGA database reveals several human cancers that have increased expression of TIGIT including colon, cervical, ovarian, head and neck squamous cell carcinoma (HNSCC), acute myeloid leukemia, multiple myeloma, and non-small cell lung cancer (NSCLC). Taken together, TIGIT has emerged as a strategic target for next generation ICB therapy. As of this writing, there are 38 clinical trials investigating the efficacy of TIGIT across a myriad of indications, as monotherapy and in combination (clinicaltrials.gov).

MC38-CEA is a murine model of colon carcinoma transduced to express carcinoembryonic antigen (CEA), a well-established self and tumor-associated antigen.^17^ Previous work completed by our group has demonstrated that the MC38-CEA colon carcinoma model is minimally responsive to monotherapy anti-PD-L1, with objective response rates of 12.5%.^18^ We therefore sought to identify rationally designed combination treatments in this model.

We show for the first time that MC38-CEA tumors are highly responsive to treatment with αTIGIT and bintrafusp alfa in combination, and this treatment strategy provides immunologic memory and protection from tumor rechallenge. This combination therapy is dependent on CD4^+^ and CD8^+^ T cells for efficacy, as well as increasing immune cell infiltration into the TME. We observe increases in numerous immune cell subsets in the TME, with increased immune activation, migration, cytotoxicity, and tumor-specific T cells. Our results in the MC38-CEA model were further confirmed through tumor control in the HPV^+^ TC1 tumor model, demonstrating our combination therapy works across multiple indications. Thus, data presented herein provide rationale for the combination of two immuno-oncology agents consisting of αTIGIT and bintrafusp alfa to enhance antitumor immunity against murine models of colorectal and HPV^+^ malignancies.

## Materials and methods

### Experimental reagents

αTIGIT (anti-muTIGIT, 18G10) is a murinized IgG2a monoclonal antibody targeting TIGIT. Delivery of αTIGIT, unless otherwise noted, is 125 µg administered intraperitoneally (*i.p*.) on days 7, 14, and 21. Bintrafusp alfa is a bifunctional fusion protein composed of the extracellular domain of the human transforming growth factor β receptor II (TGFβRII or TGFβ ”trap”) fused via a flexible linker to the C-terminus of each heavy chain of an IgG1 antibody blocking anti-PD-L1.^6,19,20^ Delivery of bintrafusp alfa, unless otherwise noted, is 492 µg delivered *i.p*. on days 7, 9, and 11. The dose used in murine studies of αTIGIT and bintrafusp alfa are the human equivalent of 5 mg/kg and 20 mg/kg, respectively. αTIGIT and bintrafusp alfa were obtained from EMD Serono (Rockland, Massachusetts, USA) through a Cooperative Research and Development Agreement (CRADA) with the National Cancer Institute (NCI), and National Institutes of Health (NIH) (Bethesda, Maryland, USA).

### Cell lines

MC38-CEA is a murine model of colon carcinoma transduced to express carcinoembryonic antigen (CEA), a well-established self and tumor-associated antigen^17^ and were generated and maintained in our laboratory as previously described.^21^ The TC1 cell line was a gracious gift from Dr. T.C. Wu (Johns Hopkins University; Baltimore, Maryland, USA).^22^ All cell lines were passaged less than 6 months, confirmed *Mycoplasma* free, and cultured at 37°C with 5% CO_2_.

### Animals and tumor models

Mice were housed in microisolator cages under specific pathogen-free conditions. A breeding pair of C57BL/6 CEA-transgenic (Tg) mice were graciously provided by Dr. John Shively (Beckman Research Institute; City of Hope National Medical Center, Duarte, California, USA) and were bred and maintained at the National Institutes of Health. These animals are homozygous for CEA and are used as a self-antigen model.^23,24^ All animal studies were approved and conducted in accordance with an Institutional Animal Care and Use Committee (IACUC)‒approved animal protocol (#LTIB-38 and #LTIB-57), and utilizing ARRIVE reporting guidelines.^25^

For the MC38-CEA model, 8–16-week-old female C57BL/6 CEA-Tg (referred to as CEA.Tg henceforth) mice were inoculated with 3 × 10^5^ MC38-CEA tumor cells subcutaneously (*s.c*.) in the right flank. For the TC1 model, 8–16-week-old female C57BL/6 mice were inoculated with 5 × 10^4^ TC1 tumor cells subcutaneously in the right flank. Treatment initiation occurred on day 7, or when mean tumor volume was between 50 and 100mm^3^. Where indicated, MC38-CEA and TC1 tumor-bearing mice were treated three times with 125 µg αTIGIT, delivered *i.p*. one week apart, and three doses of 492 µg bintrafusp alfa *i.p*., every other day (graphical representation of experimental design, see Figure 2a and Figure 6a). For depletion studies, anti-CD4 (GK1.5, 100 µg; BioXcell; Lebanon, New Hampshire, USA) and anti-CD8 (2.43, 100 µg; BioXcell) antibodies were administered *i.p*. on days 3, 4, 5, 12, 19, and 26 post-tumor inoculation. The anti-NK1.1 (PK136, 100 µg; BioXcell) antibody was administered *i.p*. on days 3, 4, 5, 8, 11, 14, 17, 20, 23, and 26 post-tumor inoculation (graphical representation of experimental design, see Figure 3a). For tumor rechallenge studies, naïve control mice and previously challenged tumor-free mice were inoculated with 1 × 10^6^ MC38-CEA cells *s.c*. in the opposite flank 60 days after initial tumor inoculation, roughly 40 days after cessation of treatment (graphical representation of experimental design, see Figure 2a).

For all animal tumor studies, tumor growth was monitored biweekly and animal weight at least once per week. Termination of studies is indicated in figures, or when animals reached ethical limit (2000 mm^3^, or 20 mm in length or width).

### Serum cytokine analyses

Serum collection was performed on day 0, prior to tumor inoculation, and at indicated time points (*i.e*., day 14 and end of study). Serum IL-1β, IL-2, IL-5 and TNFα were quantified using the murine V-Plex Proinflammatory Panel 1 kit and MESO QuickPlex SQ 120 (Meso Scale Diagnostics; Rockville, Maryland, USA), according to the manufacturer’s instructions. TGFβ levels were quantified using mouse TGFβ1 Quantikine ELISA kit according to the manufacturer’s instructions (R&D Systems; Minneapolis, Minnesota, USA). Data presented herein represent the change in cytokine, with each animal’s baseline levels subtracted from subsequent timepoints.

### RNA analyses

Total RNA was isolated from indicated tumors at day 24 post-tumor inoculation using the RNeasy Mini kit (Qiagen; Germantown, Maryland, USA). NanoString nCounter® PanCancer Profiling Panel (NanoString Technologies; Seattle, Washington, USA) analysis was performed by the Genomics Laboratory, Frederick National Laboratory for Cancer Research (Frederick, Maryland, USA). Raw data (RCC) files were uploaded into nSolver analysis software. Treated samples were compared with control samples, and ratio fold-change data were exported to GraphPad Prism (San Diego, California, USA).

Common genes associated with immune cell adhesion/migration, immune activation, immune regulation, matrix remodeling/metastasis, myeloid compartment, cytokine and chemokine, tumor progression and tumor suppression as identified by NanoString and current literature are denoted. Common genes associated with cellular signaling pathways as identified by NanoString and current literature are denoted. Select curated genes were chosen based on NanoString data and included in a STRING analysis of protein–protein interactions (string-db.org;).^26^

### ELIspot

Spleens were harvested and processed individually into single-cell suspensions. 1 × 10^6^ splenocytes were plated onto 96-well plates previously coated with an IFNγ capture antibody (BD Cat# 551083). C57BL/6-CEA Tg splenocytes were stimulated with one of the following  H2-D^b^- or H2-K^b^-restricted peptides (10 µg/mL) for 18 hours: CEA_526-533_ (EAQNTTYL), CEA_572-579_ (GIQNSVSA), p15E (KSPWFTTL), and HIV-gag (SQVTNPANI). The CEA_526-533_, CEA_572-579_, p15E, and HIV-gag peptides were synthesized by CPC Scientific (Sunnyvale, California, USA). IFNγ spots were detected using the BD mouse IFNγ ELISPOT kit and developed using the BD ELISPOT AEC substrate set according to the manufacturer’s instructions (BD Biosciences; San Jose, California, USA). IFNγ spots were visualized and quantified using the CTL ImmunoSpot Analyzer (Cleveland, Ohio, USA).

### Flow cytometry

Tumors were excised and mechanically dissociated to generate single-cell suspensions. Total cell number and viability (trypan blue) were calculated using the Cellometer 2000 (Nexcelom; Lawrence, Massachusetts, USA). The following murine antibodies from BioLegend (San Diego, California, USA) were used for flow cytometric staining: CD155-APC (Clone # TX56), PD-L1-BV785 (Clone # 10 F.9G2), Ly6G-BV421 (Clone # 1A8), CD11b-BV510 (Clone # M1/70), CD11c-APC-Cy7 (Clone # N418), F4/80-BV605 (Clone # BM8), CD3-PE-Cy5 (Clone # 145–2C11), CD226-FITC (Clone # 10E5), CD62L-BV785 (Clone # MEL-14), PD1-PE-Cy7 (Clone # RMP1-30), CD44-AF700 (Clone # IM7), CD4-PE-Cy5 (Clone # RM4-5), and CD155-APC (Clone # TX56). The following antibodies from BD Biosciences (Franklin Lakes, New Jersey, USA) were used for flow cytometric staining: CD226-BV650 (Clone # 10E5), Ly6C-FITC (Clone # AL-21), CD49b-BUV395 (Clone # HMα2), TIGIT-BV711 (Clone # 1 G9), B220-BV711 (Clone # RA3-6B2), LAG3-BV605 (Clone # C9B7W), CD45-PE (Clone # 30-F11). The following antibodies from Invitrogen (Waltham, Massachusetts, USA) were used for flow cytometric staining: CD8-SB645 (Clone # 53–6.7) and FoxP3-PE-Cy5.5 (Clone # FJK-16s). Live/Dead fixable aqua stain set was purchased from Thermo Fisher (Waltham, Massachusetts, USA). Intracellular staining was performed using the FoxP3/transcription factor kit (eBioscience; San Diego, California, USA). Cytometric data were acquired on a BD LSRFortessa (BD Biosciences) and analyzed using FlowJo 10.7.1 (TreeStar; Ashland, Oregon, USA).

Cell populations were gated on FSC × SSC discrimination, live:dead, CD45+ and then as followed: Regulatory T cells (CD3+, CD4+, FoxP3+); CD4+  T cells (CD3+, CD4+, FoxP3-); CD8+  T cells (CD3+, CD4-, CD8+); NK cells (CD3-, CD49b+); monocytic myeloid-derived suppressor cells (M-MDSCs) (CD3-, CD49b-, CD11b+, Ly6g-, Ly6C+); polymorphonuclear MDSCs (PMN-MDSCs) (CD3-, CD49b-, CD11b+, Ly6G+, Ly6C-); macrophages (CD3-, F4/80+, CD11b+) and DCs (CD3-, CD49b-, F4/80, CD11c+).

### Statistical analyses

Student *t* test was used to compare two groups. One-way or two-way ANOVA was performed to compare more than two groups with Tukey’s post hoc analysis for correction. Log-rank (Mantel-Cox) test was used to determine survival proportions. Two-way ANOVA was performed to compare significant gene changes. *P* values less than 0.05 were considered significant with * = *p* < .05, ** =* p* < .01, *** =* p* < .005, *** =* p* < .0001. Error bars in figures represent mean ± SEM. GraphPad Prism 9.0 was utilized for analyses.

## Results

### TIGIT, PD-1, and TGFβ are candidate targets for combination therapy in the MC38-CEA murine colorectal tumor model

**Figure 1.**
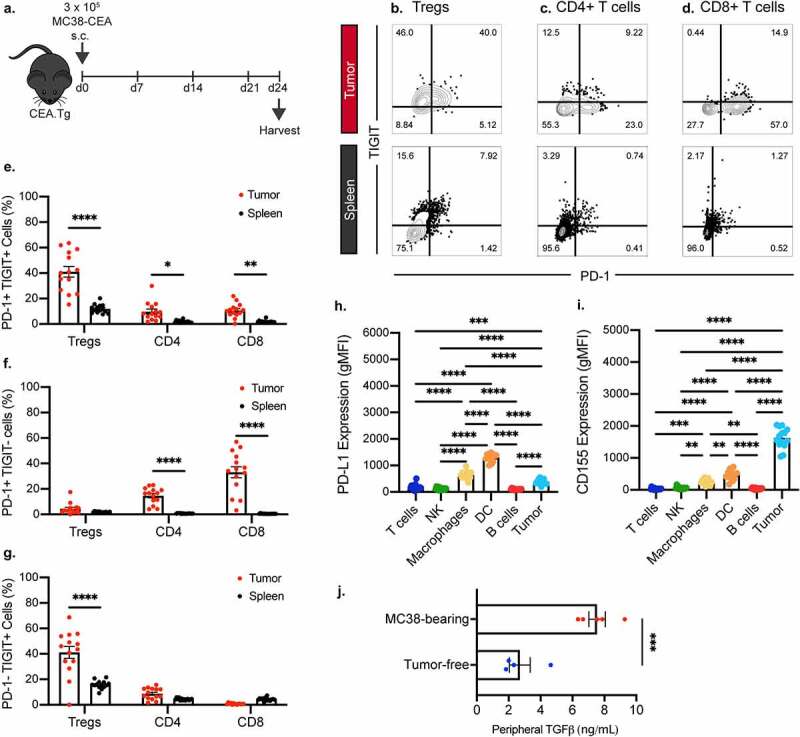
TIGIT and PD-1 are significantly upregulated on tumor infiltrating CD4^+^, regulatory, and CD8^+^ T cells while TGFβ level is increased in plasma of tumor-bearing mice. (a) Graphical representation of experimental design. Representative flow cytometric plots of frequency of TIGIT and PD-1 positivity on tumor infiltrating (*n* = 14) and splenic (*n* = 14) (b) regulatory T cells, (c) CD4^+^ T cells, and (d) CD8^+^ T cells. Quantification of the frequency of (e) PD-1+ TIGIT+, (f) PD-1+ TIGIT-, (g) PD-1- TIGIT+ regulatory, CD4^+^, and CD8^+^ T cells infiltrating into the tumor (red symbols) and those found in the periphery (black symbols). Frequency is calculated on percent of parent populations. Quantification of expression levels of (h) PD-L1 and (i) CD155 on tumor and tumor-infiltrating immune cells. (j) ELISA quantification of plasma TGFβ levels in MC38 tumor-bearing mice 24 days post-tumor inoculation. * = p < .05, ** = p < .01, *** = p < .005, **** = p < .0001.

Traditional ICB therapy exploits inhibitory receptors that are upregulated on tumor infiltrating lymphocytes (TIL). However, not all TILs express these canonical exhaustion markers that are subsequently targeted using monoclonal antibodies (mAbs). To determine feasible targets in our murine models, we employed the MC38-CEA tumor cell line. MC38-CEA, a murine colon carcinoma cell line transduced to express CEA, has little responsiveness to checkpoint blockade therapy.^1^ Female CEA.Tg mice were inoculated with MC38-CEA cells and resulting tumors and spleens were harvested 24 days after instillation (Figure 1a). Expression of TIGIT and PD-1 on TILs, as well as peripheral lymphocytes in the spleen, were assessed (Figure 1b-d). Compared to peripheral lymphocytes, a significant subset of tumor infiltrating Tregs (p < .0001), CD4^+^ (p < .05), and CD8^+^ (p < .01) T cells co-express PD-1 and TIGIT (Figure 1b-d, quantified in Figure 1e). PD-1^+^ CD4^+^ (p < .0001) and CD8^+^ (p < .0001) T cells that do not co-express TIGIT were more abundant in the tumor than in the spleen while no significant changes in the frequency of PD-1^+^ TIGIT^−^ subset were observed in the Treg compartment (Figure 1b-d, quantified in Figure 1f). The frequencies of PD-1^−^ TIGIT^+^ CD4^+^ and CD8^+^ remained unchanged between the tumor and the periphery whereas the frequency of Tregs that were single positive for TIGIT was significantly higher in the tumor than the spleen (p < .0001; Figure 1b-d, quantified in Figure 1g).

Co-inhibitory receptors require cognate ligand binding to exert suppressive functions. We next investigated the expression of TIGIT and PD-1 ligands, CD155^11^ and PD-L1,^27^ respectively. Flow cytometric analysis demonstrated that in the TME, the DCs had the highest PD-L1 positivity, followed by macrophages and tumor cells (Figure 1h). Meanwhile, CD155 expression was highest in the MC38 tumor cells, with significant expression in the macrophages and DCs as well (Figure 1i).

In addition to co-inhibitory ligands, soluble immunosuppressive factors, such as TGFβ, are produced in the TME to dampen the immune response.^28^ We examined the TGFβ levels in the peripheral blood of tumor-bearing and tumor-free animals. Tumor-bearing mice had significant 3.5-fold increase in peripheral TGFβ (p < .005) compared to their tumor-free counterparts (Figure 1j).

Our data indicate that intratumoral effector T cells in the MC38-CEA model are likely inhibited through the TIGIT/CD155, PD-1/PD-L1, and TGFβ pathways. Therefore, we sought to rationally design a combination treatment strategy to disrupt these axes of immune suppression through use of a mAb targeting TIGIT (αTIGIT) and a bifunctional molecule that traps TGFβ and blocks PD-L1, bintrafusp alfa.^6,19,20^

### αTIGIT and bintrafusp alfa combination therapy resulted in significant antitumor activity, improved survival, and increased immunologic memory in the MC38-CEA tumor model

**Figure 2.**
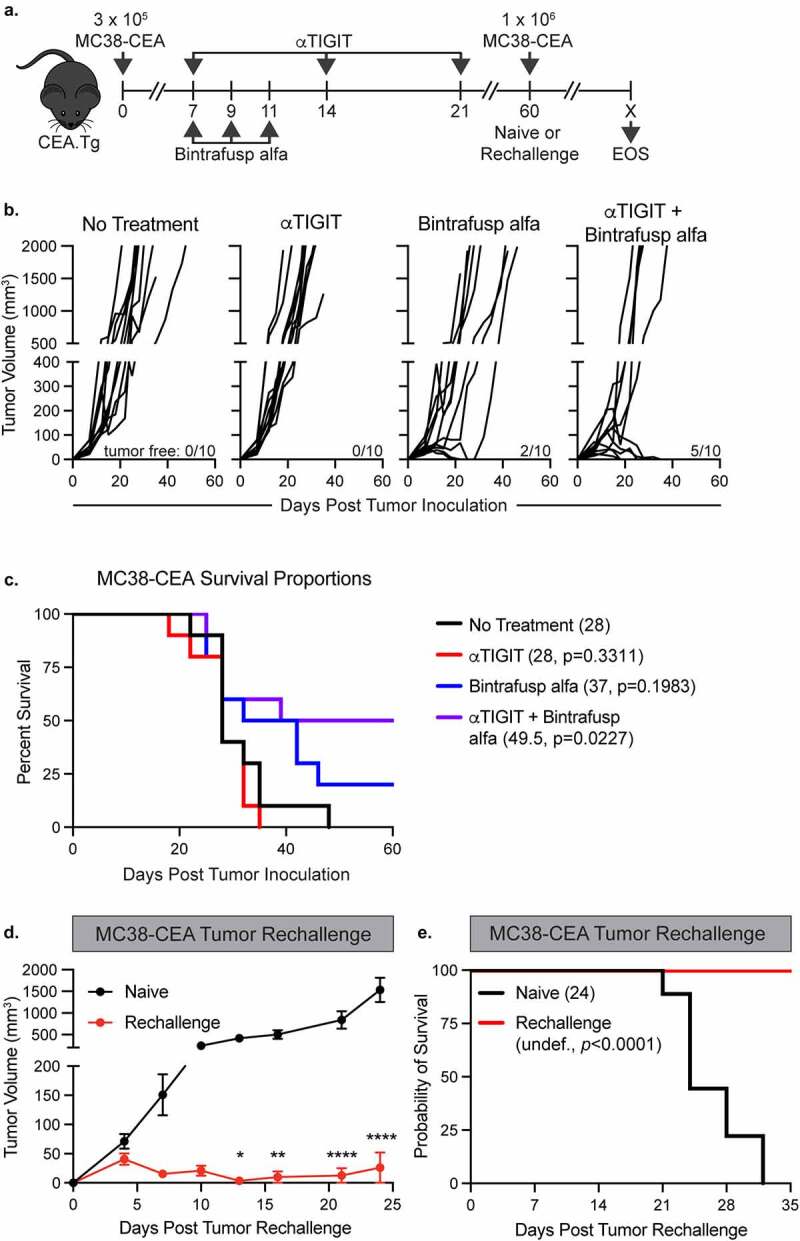
αTIGIT and bintrafusp alfa combination therapy provides significant antitumor activity, increases survival, and provides immunologic memory. (a) Graphical representation of experimental design for MC38-CEA tumor studies. (b) MC38-CEA tumor growth curves and (c) survival proportions of CEA.Tg mice treated with αTIGIT (red line; *n* = 10), bintrafusp alfa (blue line; *n* = 10), αTIGIT + bintrafusp alfa (purple line; *n* = 10) or untreated controls (black line; *n* = 10). Numbers in parentheses indicate median overall survival in days. Tumor-free mice (red line; *n* = 7) from (c) were rechallenged with MC38-CEA cells and monitored for (d) tumor progression and (e) overall survival in comparison to naïve mice (black line; *n* = 10).

We first sought to determine the *in vivo* efficacy of αTIGIT + bintrafusp alfa in combination utilizing the MC38-CEA tumor model (Figure 2a). αTIGIT had no antitumor activity when utilized as monotherapy (Figure 2b), while treatment with bintrafusp alfa alone resulted in 20% of the animals being tumor free. Treatment with the combination of αTIGIT and bintrafusp alfa, however, did significantly control tumors (p = .0073; Figure 2b). Fifty percent of animals treated with αTIGIT + bintrafusp alfa were tumor-free 60 days post-tumor inoculation (Figure 2b and 2c). This combination treatment resulted in increased overall survival of MC38-CEA tumor-bearing mice (Figure 2c), extending survival from 28 days in untreated controls to 49.5 days in animals treated with αTIGIT + bintrafusp alfa (p = .0227; Figure 2c). Treatment with either αTIGIT or bintrafusp alfa alone did not result in a significant increase in overall survival.

Because we observed 50% of animals treated with αTIGIT + bintrafusp alfa can eliminate MC38-CEA tumors, we next determined if this combination therapy provided protection from tumor rechallenge. Tumor-free animals were inoculated with an MC38-CEA tumor cell burden 3.5 times higher than the initial challenge in the opposite flank and tumor growth was monitored biweekly (Figure 2a). Animals that had previously received αTIGIT + bintrafusp alfa were protected from tumor rechallenge, with significantly reduced tumor volume (p < .0001; Figure 2d). All animals facing rechallenge were protected from lethal tumor burden, as indicated by an increased overall survival in comparison to naïve control mice (Figure 2e). These data indicate that αTIGIT + bintrafusp alfa provide immunologic memory, allowing animals that initially responded to treatment to remain tumor free following high-dose rechallenge.

To determine if both components of the bifunctional bintrafusp alfa were required for the antitumor activity observed, we compared a mutated version of the molecule, where sequestration of TGFβ is maintained but binding of PD-L1 is abrogated (online supplemental figure 1A; designated bintrafusp alfa-Mut). Consistent with previous results, monotherapy treatment with αTIGIT or bintrafusp alfa had no antitumor activity, while αTIGIT + bintrafusp alfa combination therapy resulted in significant antitumor activity compared to the control cohort (p = .0259) with 50% of mice rendered tumor free at the end of the study (online supplemental figure 1B). Importantly, mice treated with the combination of αTIGIT + bintrafusp alfa-Mut did not control tumor burdens, indicating that both sequestration of TGFβ and binding of PD-L1 at the tumor site are critical. These results are consistent with current literature.^29^

### Antitumor activity and increase in overall survival from treatment with αTIGIT and bintrafusp alfa are dependent on CD4^+^ and CD8^+^ T cells

**Figure 3.**
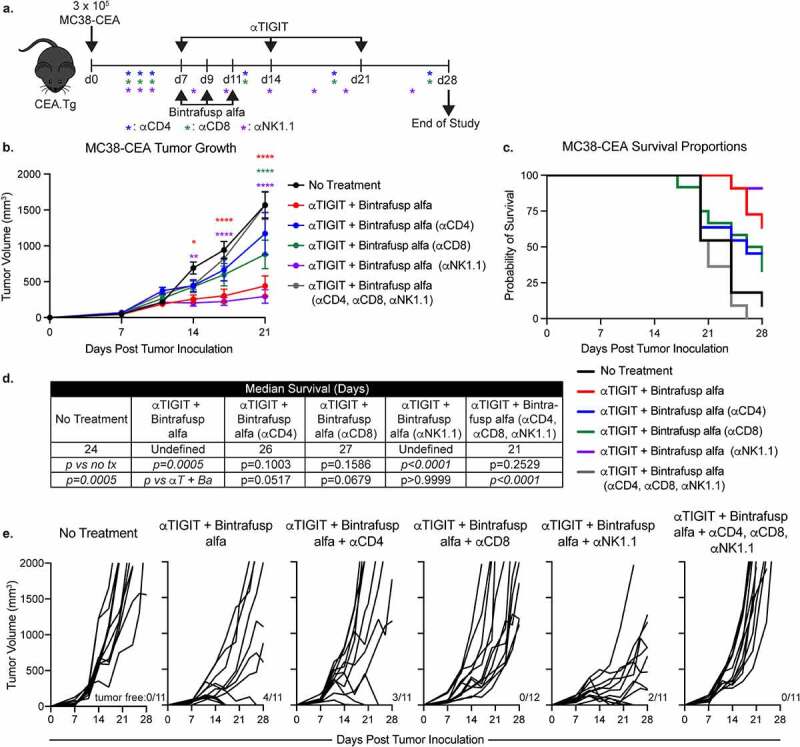
Antitumor activity and increase in overall survival from treatment with αTIGIT and bintrafusp alfa is dependent on CD4^+^ and CD8^+^ T cells. (a) Graphical representation of experimental design. (b) MC38-CEA tumor growth curves and (c) survival proportions of CEA.Tg animals treated with αTIGIT + bintrafusp alfa (red line; *n* = 11), αTIGIT + bintrafusp alfa depleted of CD4**^+^** T cells (blue line; *n* = 11), αTIGIT + bintrafusp alfa depleted of CD8**^+^** T cells (green line; *n* = 12), αTIGIT + bintrafusp alfa depleted of NK cells (purple line; *n* = 11), αTIGIT + bintrafusp alfa depleted of CD4**^+^**, CD8**^+^** and NK cells (gray line; *n* = 11), and untreated animals (black line; *n* = 11). (d) Median survival of each treatment group. (e) Individual animal tumor growth rates of each treatment group. Numbers at the bottom right of tumor growth rate plots indicate tumor-free mice. * = p < .05, ** = p < .01, *** = p < .005, *** = p < .0001.

ICB therapy can exert its effects on immune cells, tumor cells directly, or a combination of the two.^4^ To identify the cell types required for the antitumor activity observed with αTIGIT and bintrafusp alfa combination therapy we utilized immune cell depletion studies. MC38-CEA tumor-bearing mice were depleted separately of CD4^+^ T cells, CD8^+^ T cells, NK cells, or all in combination, while simultaneously receiving αTIGIT and bintrafusp alfa (Figure 3a). Consistent with our previous studies, animals in the αTIGIT + bintrafusp alfa cohort had significantly reduced tumor volume in comparison to untreated control mice (p < .0001; Figure 3b, red line; Figure 3e), as well as an increased frequency of tumor-free mice at end of study (4/11; Figure 3e). When the CD4^+^ T cell compartment was depleted, the antitumor activity provided by our doublet therapy was lost, as indicated by similar tumor volume to untreated mice (Figure 3b, blue line; Figure 3e). Depletion of the CD8^+^ T cell compartment resulted in a failure to control tumor volumes early in tumor progression, although not to the extent as the untreated group by day 21 (p < .0001; Figure 3b, green line; Figure 3e). TIGIT expression is highest on T cells and NK cells, and it was therefore unexpected that the antitumor activity observed with combination treatment of αTIGIT and bintrafusp alfa was upheld after depletion of NK cells (p < .0001; Figure 3b, purple line; Figure 3e). To determine if there were additional cell types that contribute to the effect of αTIGIT and bintrafusp alfa combination treatment, MC38-CEA tumor bearing animals were depleted of CD4^+^, CD8^+^ and NK cells (gray line), which resulted in rapid tumor progression (Figure 3 b and e). The survival advantage previously observed with our combination treatment was abrogated following depletion of CD4^+^ T cells (blue line), CD8^+^ T cells (green line) and combination of CD4^+^, CD8^+^ and NK cells (gray line) (Figure 3 c and d). Increase in overall survival was upheld in animals treated with αTIGIT and bintrafusp alfa combination therapy (red line), and when NK cells were depleted (purple line) (Figure 3 c and d). The median survival of CD4^+^ T cell and CD8^+^ T cell-depleted groups was less in comparison to the αTIGIT and bintrafusp alfa group (p = .0517 and p = .0679, respectively; Figure 3d). These data suggest the mechanism of action exerted by this combination therapy lies within the T cell compartment, with little contribution from NK cells.

### αTIGIT and bintrafusp alfa combination treatment increases immune cell infiltration to the tumor microenvironment

**Figure 4.**
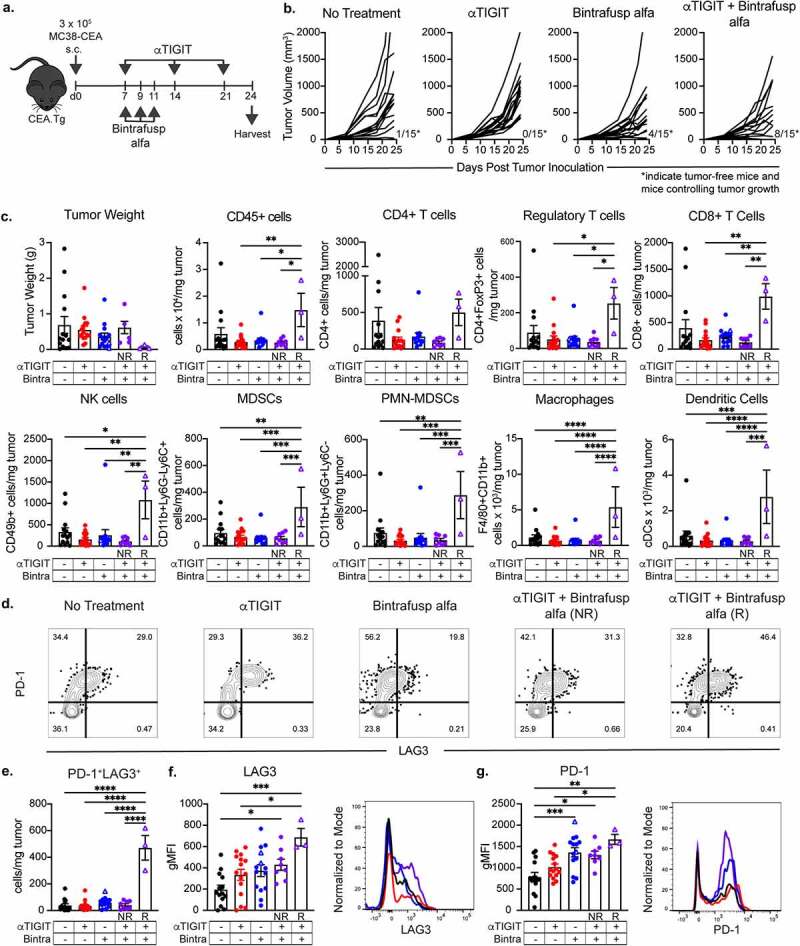
αTIGIT and bintrafusp alfa combination treatment increases immune cell infiltration to the tumor microenvironment. (a) Graphical representation of experimental design. (b) MC38-CEA tumor growth curves of CEA.Tg animals treated with αTIGIT (*n* = 15), bintrafusp alfa (*n* = 15), αTIGIT + bintrafusp alfa (*n* = 15) or untreated animals (*n* = 15). Numbers at bottom right of graphs indicate tumor-free mice and mice responding to therapy that possess tumors less than 300mm^3^ at end of study (termed ‘Responders’). (c) At day 24 post tumor inoculation, tumors from mice treated with αTIGIT (*n* = 15), bintrafusp alfa (*n* = 14), αTIGIT + bintrafusp alfa Non-Responders (*n* = 8), αTIGIT + bintrafusp alfa Responders (*n* = 3) and untreated animals (*n* = 14) were excised and tumor weight, CD45**^+^** cells/mg of tumor, and indicated immune cell subsets were quantified via flow cytometry and normalized to recorded tumor weight. (d) Gated on CD8**^+^** T cells, evaluation of PD-1 and LAG3 dual positive cells from indicated treatment groups. (e) Quantification of PD-1**^+^**LAG3**^+^** CD8**^+^** T cells per mg of tumor. Quantification of expression levels of (f) LAG3 and (g) PD-1 on CD8**^+^** T cells and representative histograms from MC38-CEA tumor-bearing mice treated with αTIGIT (red line), bintrafusp alfa (blue line), αTIGIT + bintrafusp alfa Responders (purple line) and untreated (black line). NR = Non-Responders (mice with tumor volumes greater than 300mm^3^ at day 24, closed symbols). R = Responders (mice with tumor volumes less than 300mm^3^ at day 24, open triangles). Bintra = bintrafusp alfa. gMFI = geometric mean fluorescence intensity. * = p < .05, ** = p < .01, *** = p < .005, *** = p < .0001.

One of the failures of checkpoint blockade therapy can be attributed to an inability of immune cells to access the TME and, consequently, an inability to exert effector function.^30^ We therefore sought to determine the effect(s) of combination treatment with αTIGIT and bintrafusp alfa on the TME. Because we observed 50% of animals treated with αTIGIT and bintrafusp alfa in combination were tumor free at the end of study, we next interrogated factors associated with response or resistance to therapy. MC38-CEA tumor-bearing mice were treated with αTIGIT and bintrafusp alfa, alone and in combination (Figure 4a). At end of study, animals treated with αTIGIT and bintrafusp alfa had significantly smaller tumor volumes in comparison to untreated animals (p < .0001), and those that received either agent as monotherapy (Figure 4b). Tumor volume was monitored biweekly until day 24, at which point tumors were excised and the TME was interrogated via flow cytometry. On day 24, 5/15 (33.3%) of the animals in the combination treatment group were tumor-free, 3/15 (20%) of animals were controlling tumor growth, as indicated by tumors smaller than 300 mm^3^, and 7/15 (46.7%) animals were incapable of controlling tumor growth. Heterogeneity in responses to ICB by genetically identical tumor-bearing mice has been reported and utilized to identify possible biomarkers that can be used to predict response to ICB.^31,32^ Hence, the αTIGIT and bintrafusp alfa combination treatment group was then further categorized into animals that responded to therapy (~54%) [termed ‘Responders’ – open purple triangles (R)] and those that were resistant to therapy (~46%) [termed ‘Non-Responders’ – closed purple circles (NR)]. We observed the smallest tumors by volume and weight from the αTIGIT + bintrafusp alfa Responders cohort, which also had the greatest infiltration of immune cells into the tumor (Figure 4c). This group had significant increases in CD45^+^ cells, regulatory and CD8^+^ T cells, NK cells, M-MDSCs, PMN-MDSCs, macrophages, and DCs (Figure 4c). Due to the global increase in all immune cell subsets queried, there was not a significant increase in the CD8:Treg ratio between treatment groups (online supplemental figure 2D).

We observed a significant reduction in TIGIT expression on CD4^+^, regulatory, and CD8^+^ T cells, as well as NK cells when αTIGIT was administered, suggesting sufficient blocking of this receptor is achieved *in vivo* (online supplemental figure 2A). Recent evidence in melanoma patients suggests a high TIGIT:CD226 ratio on Tregs is an indicator of poor response to ICB.^33^ Indeed, we observed a significant reduction in the bioavailable TIGIT-to-CD226 ratio on tumor infiltrating Tregs in all groups receiving αTIGIT, as monotherapy or in combination (p < .0001; online supplemental figure 2C).

It has been reported previously that the therapeutic benefit of TIGIT blockade is dependent upon the sustained positive interaction of CD226 with its cognate receptor shared with TIGIT, CD155.^14^ In our model, we did not observe any substantial alterations in CD226 expression on CD4^+^, regulatory and CD8^+^ T cells, or NK cells (online supplemental figure 2B). CD155 expression was not significantly altered on M-MDSCs, PMN-MDSCs, macrophages, NK cells or DCs (online supplemental figure 2E).

Upon closer inspection of CD8^+^ T cells, we looked at expression patterns of two prototypical exhaustion markers, PD-1 and LAG3 (Figure 4d-g). Representative flow cytometric plots examining co-expression of PD-1 and LAG3 revealed a substantial population of PD-1^+^LAG3^+^ CD8^+^ T cells infiltrating the tumor in all groups (Figure 4d). However, αTIGIT + bintrafusp alfa Responders had the highest frequency of dual-positive CD8^+^ T cells co-expressing PD-1 and LAG3 per mg of tumor in comparison to all other treatment groups (p < .0001; Figure 4e). Not only were PD-1^+^LAG3^+^ CD8^+^ T cells more abundant in αTIGIT + bintrafusp alfa Responders, the expression levels of LAG3 and PD-1 were also significantly higher as determined by geometric mean fluorescence intensity (gMFI) (p = .0009), Figure 4f; and (p = .0016), Figure 4g.

### αTIGIT + bintrafusp alfa Responders have an altered peripheral cytokine and T cell landscape

**Figure 5.**
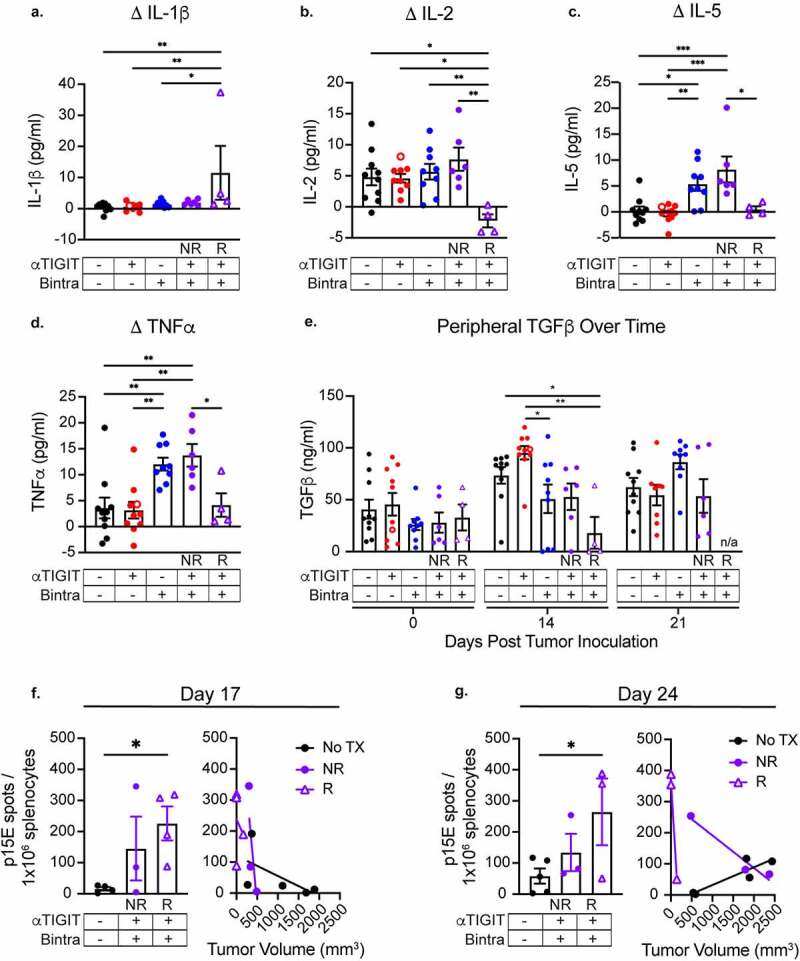
αTIGIT + bintrafusp alfa Responders have an altered peripheral cytokine and T cell landscape. Serum was collected from MC38-CEA tumor-bearing mice treated with αTIGIT (red symbols; *n* = 10), bintrafusp alfa (blue symbols; *n* = 19), αTIGIT + bintrafusp alfa Non-Responders (closed purple symbols; *n* = 6), αTIGIT + bintrafusp alfa Responders (open purple triangles; *n* = 4) and untreated animals (black symbols; *n* = 10) and assessed for change in peripheral (a) IL-1β, (b) IL-2, (c) IL-5, (d) TNFα and (e) TGFβ. Change is calculated by subtracting baseline serum levels from day 14 for IL-1β, IL-2, IL-5, and TNFα, and at day 14 and when animals reach ethical limits (tumors > 2000 m^3^) for TGFβ. Splenocytes were isolated from MC38-CEA tumor-bearing mice on (f) day 17 and (g) day 24 and p15E specific CD8**^+^** T cells were identified via ELIspot (left) and relationship to tumor volume was determined (right). Bintra = bintrafusp alfa. EOS = end of study (tumors > 2000mm^3^). NR = Non-Responders. R = Responders. No TX = No Treatment. * = p < .05, ** = p < .01, *** = p < .005, *** = p < .0001.

To investigate the role of αTIGIT + bintrafusp alfa combination treatment on host cytokine production, serum was collected from MC38-CEA tumor-bearing mice prior to tumor instillation, and at indicated times throughout the course of treatment with αTIGIT and bintrafusp alfa (Figure 5). αTIGIT + bintrafusp alfa Responders had significantly higher peripheral levels of IL-1β (p = .0093), and significantly lower levels of IL-2 in comparison to other treatment groups (Responders vs. Non-Responders p = .0014; Responders vs. No Treatment p = .0184; Figure 5a, 5b). Interestingly, αTIGIT + bintrafusp alfa Non-Responders had elevated peripheral levels of IL-5 (p = .0109) and TNFα (p = .0494) in comparison to Responders, indicating a potential mechanism of action for why this group failed to respond to therapy (Figure 5 c and d). We also observed a significant decrease in TGFβ in the cohort of animals treated with αTIGIT + bintrafusp alfa that responded to treatment at day 14 post-tumor inoculation (p = .0329), Figure 5e.

To determine if there are increased frequencies of tumor antigen-specific CD8^+^ T cells, splenocytes were isolated from MC38-CEA tumor-bearing mice on day 17 (Figure 5f) and day 24 (Figure 5g) post-tumor inoculation and co-incubated with p15E, a common MHC class I restricted retroviral protein expressed in the MC38 tumor cell line. Animals that responded to treatment with αTIGIT + bintrafusp alfa had significant increases in the number of p15E-reactive CD8^+^ T cells in comparison to untreated animals on both day 17 and day 24 (p = .0443, Figure 5f left panel; p = .0267, Figure 5g right panel). Additionally, the increased frequency of tumor-specific CD8^+^ T cells was inversely correlated with tumor volume, with the smallest tumors in the responding group possessing the most p15E-reactive T cells on day 17 and day 24 (Responders R^2^ = 0.9920), Figure 5f and 5g, right panel.

### αTIGIT and bintrafusp alfa combination therapy result in significant antitumor activity in the TC1 murine tumor model

**Figure 6.**
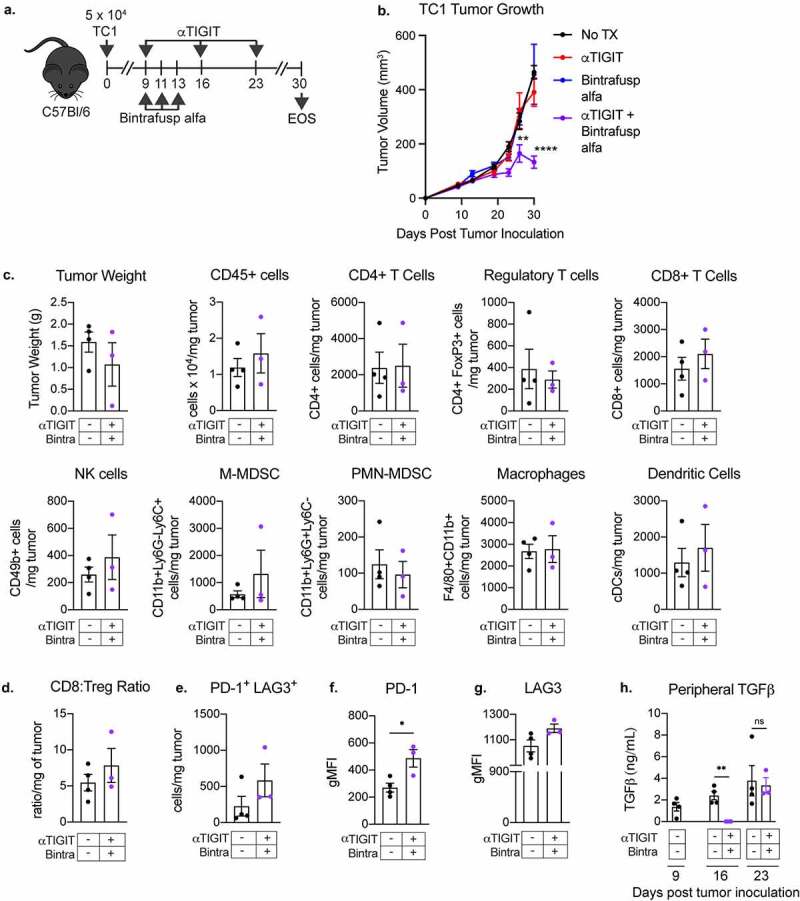
αTIGIT and bintrafusp alfa combination treatment results in antitumor activity in the TC-1 tumor model. (a) Graphical representation of experimental design for TC1 tumor studies. (b) TC1 tumor growth curves of C57BL/6 mice treated with αTIGIT (red line; *n* = 9), bintrafusp alfa (blue line; *n* = 8), αTIGIT + bintrafusp alfa (purple line; *n* = 9) or untreated controls (black line; *n* = 9). (c) A different set of mice were treated with the αTIGIT and bintrafusp alfa combination and on day 26 post tumor inoculation, tumors from mice treated with αTIGIT + bintrafusp alfa (*n* = 3) and untreated animals (*n* = 4) were excised and tumor weight and indicated immune cell subsets were quantified via flow cytometry and normalized to recorded tumor weight. (d) Quantification of CD8^+^ effector to Treg ratio. (e) Quantification of PD-1**^+^**LAG3**^+^** CD8**^+^** T cells per mg of tumor. Quantification of expression levels of (f) PD-1 and (g) LAG3 on CD8**^+^** T cells. (h) Plasma was collected from untreated and αTIGIT + bintrafusp alfa-treated TC1 tumor-bearing mice on days 9, 16, and 23 post-tumor implantation and assessed for TGFβ. Bintra = bintrafusp alfa. EOS = end of study (tumors > 2000mm^3^). gMFI = geometric mean fluorescence intensity. * = p < .05, ** = p < .01, *** = p < .005, *** = p < .0001.

As previously mentioned, TIGIT is upregulated on numerous human cancers, including human papillomavirus (HPV)‒associated malignancies. We therefore sought to determine if our combination therapy exhibits antitumor efficacy in the TC1 tumor model, a murine lung carcinoma transformed to express E6 and E7, the dominant oncolytic proteins of HPV16 (Figure 6a). At the end of study, mice that received αTIGIT and bintrafusp alfa in combination had significantly lower tumor burden in comparison to untreated mice, or mice that received αTIGIT or bintrafusp alfa as monotherapies (p < .0001; Figure 6b). Together with the MC38-CEA data, this indicates that αTIGIT and bintrafusp alfa used in combination have significant antitumor activity in two distinct tumor models.

In another set of TC1-bearing mice treated with the αTIGIT and bintrafusp alfa combination (Figure 6a), tumors growth was monitored and on day 26 tumors were excised. The average tumor volume in the cohort that received the αTIGIT and bintrafusp alfa combination was significantly smaller than that in the untreated group (online supplemental figure 3A). The sample size did not allow for the differentiation of Responders from Non-Responders, which may explain why the flow cytometric analysis of TC1 tumors treated with αTIGIT + bintrafusp alfa did not show dramatic changes in immune infiltrates as that observed in the Responders in the MC38-CEA model. Nevertheless, we observed a trending increase of more than 30% in CD45^+^ cells, CD8^+^ T cells, NK cells, M-MDSCs, and DCs (Figure 6c). Because there were only slight changes in the immune cell subsets queried, a significant increase in the CD8:Treg ratio between groups was not observed (Figure 6d).

In the TC1 tumors treated with αTIGIT and bintrafusp alfa, we observed a significant reduction in TIGIT expression on CD4^+^ T cells (p = .0123) and a trending decrease in TIGIT expression in Treg cells, which were associated with the significant reduction in the bioavailable TIGIT-to-CD226 ratio on these tumor infiltrating cells (p = .0029 and p = .0459, respectively; online supplemental figure 3B-3C). However, we did not observe any changes in the TIGIT expression on tumor infiltrating CD8^+^ T cells (online supplemental figure 3B-3C). Similar to the MC38-CEA model, we did not observe any substantial alterations in CD226 expression on CD4^+^, regulatory and CD8^+^ T cells (online supplemental figure 3B-D) and in CD155 expression on MDSCs, macrophages, or DCs (online supplemental figure 3E) in the TC1 model.

The PD-1 and LAG3 expression on CD8 + T cells were examined (Figure 6e-g). Although not statistically significant, the αTIGIT + bintrafusp alfa combination treatment doubled the population of PD-1^+^LAG3^+^ CD8^+^ T cells infiltrating the tumor (Figure 6e). In addition, the combination treatment resulted in significantly higher expression level of PD-1 as determined by geometric mean fluorescence intensity (p = .0235), Figure 6f.

Analysis of the plasma collected at different time points showed that there was an incremental increase in peripheral TGFβ in the untreated animals (Figure 6g). On day 16, which was 3 days after the last bintrafusp alfa treatment, TGFβ levels decreased significantly in the animals treated with the αTIGIT + bintrafusp alfa combination when compared to the control (p = .0033; Figure 6g). Because only one round of bintrafusp alfa was administered, peripheral TGFβ levels were back to the same level as the untreated cohort on day 23 (Figure 6g).

### Differentially expressed genes following αTIGIT + bintrafusp alfa treatment

**Figure 7.**
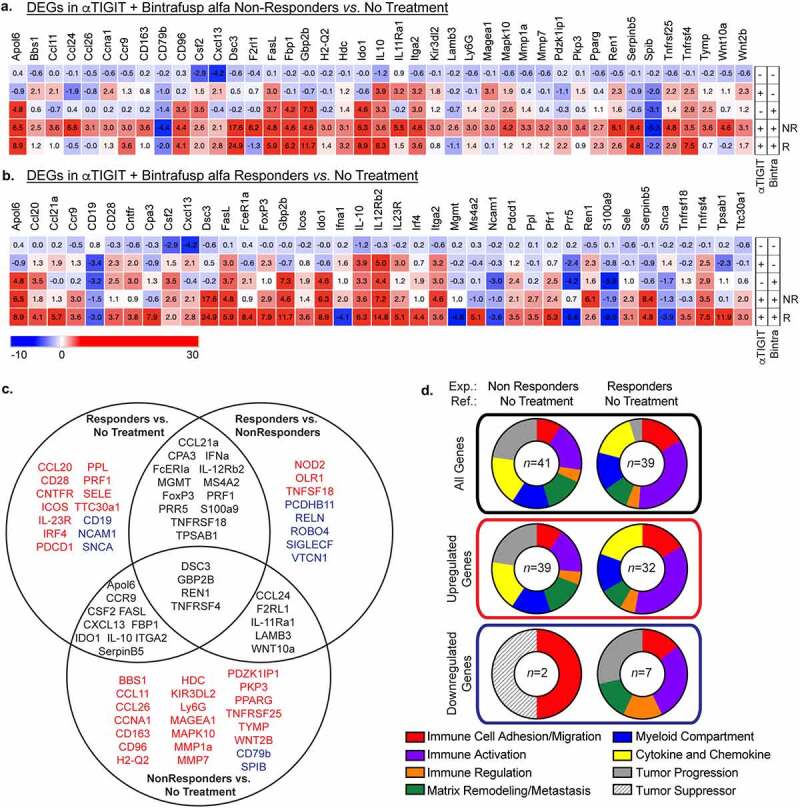
Differentially expressed genes following αTIGIT + bintrafusp alfa treatment. (a) Heatmap of significant differentially expressed genes between tumors from mice treated with αTIGIT + bintrafusp alfa Non-Responders in comparison to untreated animals as identified by the NanoString nCounter® PanCancer Pathways Panel. Mean fold change values are within each cell. (b) Heatmap of significant differentially expressed genes between tumors from mice treated with αTIGIT + bintrafusp alfa Responders in comparison to untreated mice as identified by the NanoString nCounter® PanCancer Pathways Panel. Mean fold change values are within each cell. Monotherapy treatment groups are included for reference (No Treatment *n* = 5; αTIGIT *n* = 5; bintrafusp alfa *n* = 6; αTIGIT + bintrafusp alfa Non-Responders *n* = 5; αTIGIT + bintrafusp alfa Responders *n* = 3). (c) Venn diagram of differentially expressed genes that are significant between αTIGIT + bintrafusp alfa Responders and untreated (left), αTIGIT + bintrafusp alfa Non-Responders and untreated (bottom), and αTIGIT + bintrafusp alfa Responders and αTIGIT + bintrafusp alfa Non-Responders (right). Genes in red are upregulated, genes in blue are downregulated. (d) Graphical representation of function of differentially expressed genes (top row – all genes, middle row – upregulated genes, lower row – downregulated genes) between experimental groups and reference groups. DEGs = differentially expressed genes. NR = Non-Responders. R = Responders. Exp. = experimental group. Ref. = reference group. Numbers in the middle of the pie charts indicate the number of differentially expressed genes.

To identify potential mechanism(s) of action that predict response or resistance to αTIGIT + bintrafusp alfa combination therapy, we interrogated the transcriptomic landscape within the tumor of MC38-CEA tumor-bearing mice at day 24 post-tumor inoculation. Data presented herein represent differentially expressed genes (DEGs) in αTIGIT + bintrafusp alfa Non-Responders in comparison to untreated controls (Figure 7a), and DEGs in αTIGIT + bintrafusp alfa Responders in comparison to untreated controls (Figure 7b). The full list of DEGs that are altered at least 2-fold in either direction in comparison to the untreated control cohort is depicted in Figure 7c. We generated eight categories the DEGs fell under as they pertain to the immune profile, including genes associated with immune cell adhesion/migration (red), immune activation (purple), immune regulation (orange), matrix remodeling/metastasis (green), the myeloid compartment (blue), cytokine and chemokine (including receptors/ligands/soluble factors; yellow), tumor progression (gray), and tumor suppressors (white striped) (Figure 7d). When comparing DEGs between Non-Responders and No Treatment cohorts, the DEGs fell into the following categories in order of greatest to least prevalence: tumor progression (23%), cytokine and chemokine (18%), immune activation (18%), matrix remodeling/metastasis (14%), the myeloid compartment (14%), immune cell adhesion/migration (9%), and genes involved in immune regulation (4%) (Figure 7d, left side, top panel). Of these total genes, all DEGs were upregulated except for two, CD79b and Spib (Figure 7a). Of the DEGs in Responders versus No Treatment, the DEGs fell into the following categories in order of greatest to least abundance: immune activation (35%), immune cell adhesion/migration (16%), cytokine and chemokine (16%), the myeloid compartment (14%), matrix remodeling/metastasis (9%), tumor progression (7%), and immune regulation (5%) (Figure 7d, right side, top panel). Of total DEGs, upregulated genes in Responders versus No Treatment were found in immune activation (36%), cytokine and chemokine (19%), immune cell adhesion/migration (17%), the myeloid compartment (14%), with immune regulation and matrix remodeling/metastasis each making up percentages of less than 10% (Figure 7d; right side, middle panel). Importantly, the genes that support tumor progression were all downregulated in this cohort (Figure 7d; right side, bottom panel).

**Figure 8.**
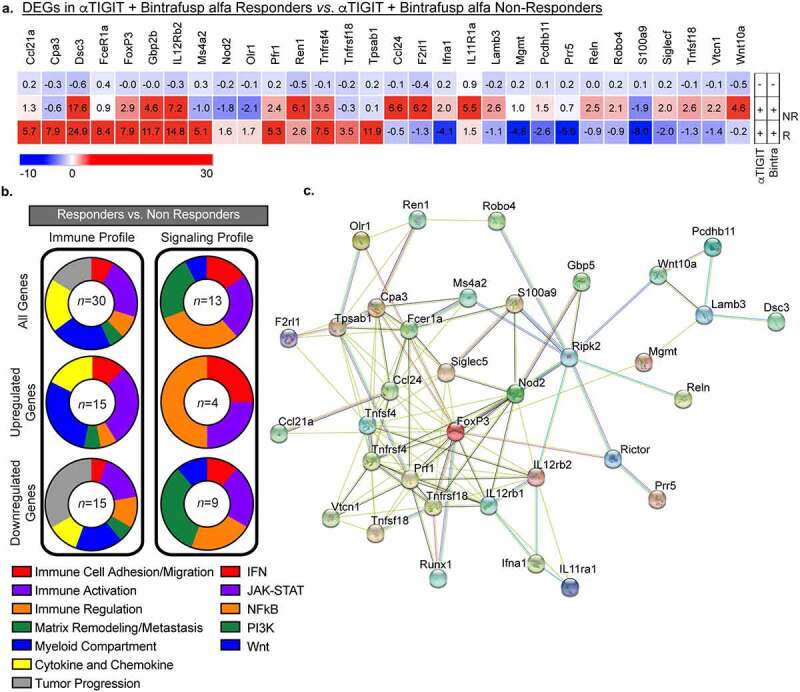
Differentially expressed genes between Responders and Non-Responders. (a) Heatmap of differentially expressed genes in tumors taken from animals treated with αTIGIT + bintrafusp alfa Non-Responders (*n* = 5), αTIGIT + bintrafusp alfa Responders (*n* = 3) in comparison to untreated animals (*n* = 5) as identified by the NanoString nCounter® PanCancer Pathways Panel. Mean fold change values are within each cell. This list represents DEGs from Responders that are statistically significant from untreated animals and Responders versus Non-Responders. (b) Graphical summary of function of DEGs (Left column – immune profile, right column – signaling profile) between Responders and Non-Responders. All genes are in the top row, upregulated genes in the middle row and downregulated genes are in the bottom row. Numbers in the middle of the pie charts indicate the number of differentially expressed genes. (c) STRING network of protein–protein interactions of DEGs between Responders and Non-Responders identified by NanoString.

Abbreviations: DEGs, differentially expressed genes; NR, Non-Responders; R, Responders.

While valuable information is gained by looking at DEGs between Non-Responders and Responders versus untreated controls, it is also important to identify differences that exist between Responders and Non-Responders treated with αTIGIT and bintrafusp alfa. DEGs presented in this heat map are significantly different between Responders versus Non-Responders, and significant to untreated controls (*at least* p < .05; Figure 8a). As performed in Figure 7d, we grouped DEGs associated with alterations in the immune profile into eight different categories (Figure 8b, left side; online supplemental table 1) and DEGs associated with five prominent signaling pathways (Figure 8b, right side; online supplemental table 2). Of all DEGs in Responders versus Non-Responders, 23% are involved in immune activation, 23% are found in the myeloid compartment, 20% in cytokine and chemokine, 17% in tumor progression, 8% in immune cell adhesion/migration, 8% immune regulation, and 6% in matrix remodeling/metastasis (Figure 8b, left side, top panel). Of the genes that are upregulated, 30% relate to immune activation, 30% in the myeloid compartment, 17% in cytokine and chemokine, 12% in immune cell adhesion/migration, with immune regulation and matrix remodeling/metastasis accounting for 6% each (Figure 8b, left side, middle panel). The most significant proportion of downregulated genes in Responders versus Non-Responders reside in the tumor progression category, making up 34% of all downregulated DEGs in this group, with the next largest groups being immune activation (17%) and the myeloid compartment (17%) (Figure 8b, left side, bottom panel).

When we queried differences in signaling pathways between Responders and Non-Responders, we observed significant alterations in the IFN (15%; red), JAK-STAT (23%; purple), NFκB (31%; orange), PI3K (23%; green), and Wnt pathways (8%; blue) (Figure 8 a and b, right side, top panel). Twenty-five percent of the upregulated signaling DEGs belong to the IFN and JAK-STAT pathways, and 50% to the NFκB pathway (Figure 8b; right side, middle panel). Of the downregulated DEGs, they comprise a relatively equivalent representation of the IFN (11%), JAK-STAT (22%), NFκB (22%), PI3K (33%), and Wnt (11%) pathways (Figure 8b, right side, bottom panel).

A STRING network of protein–protein interactions of a curated set of modulated genes identified through NanoString shows interconnectedness, albeit with multiple different nodes (Figure 8c). Of note, multiple genes display heavy interactions, while others are more distantly related. Future studies will aim to determine which of these players are crucial in driving response to αTIGIT + bintrafusp alfa combination therapy, and which are more indicative of failure to respond. Ideally, some of these soluble factors could be used in the clinic as biomarker(s) of success, or failure, in patients. These data indicate that not only do we observe an influx of lymphocytes into the TME, but also these cells are more activated via RNA transcriptomic profiles.

## Discussion

Treatment with ICB as monotherapy yields low durable responses; therefore, the rationale for combining ICB with next generation checkpoint molecules, cytokines, and/or cancer vaccines will likely become standard of care in several indications.^34,35^ The PD-1/PD-L1 axis is the most targeted pathway in cancer immunotherapy, with 4,400 trials opened since 2017. In 2020, 90% of new trials utilizing PD-1/PD-L1 inhibitors have been in combination studies.^13^ In our model of murine colon carcinoma, we demonstrate that the inhibitory receptors PD-1 and TIGIT are upregulated in the tumor infiltrating Tregs, CD4^+^ and CD8^+^ T cells and their respective cognate ligands, PD-L1 and CD155, are expressed in the TME (Figure 1). Our observations were similar to those reported in humans, wherein TIGIT and PD-1 are coordinately expressed on CD8^+^ T cells of cancer patients, including those with melanoma,^36^ hepatocarcinoma,^37^ head and neck squamous cell carcinoma,^38^ non-small cell lung cancer,^39^ and B-cell non-Hodgkin lymphoma.^40^ *Ex vivo* dual blockade of these two inhibitory receptors was demonstrated to restore the function of the CD8^+^ T cells,^36,37,41^ including the *in vitro* proliferation and cytokine production of tumor antigen-specific T cells.^36^ In addition to inhibitory receptors, we also observed that TGFβ levels are elevated in tumor-bearing mice (Figure 1). TGFβ is historically known to be pro-tumorigenic in nature and has resulted in the development of several small molecule inhibitors targeting this pathway for use in the clinic.^28^ TGFβ can suppress the host immune response to cancer in a myriad of ways, including, but not limited to, promotion of angiogenesis and epithelial to mesenchymal transition, impairment of CD8^+^ T cells and NK cells, reducing T cell infiltration to the tumor, and recruitment of M2 macrophages and MDSCs. Overall, our findings provide justification for combining two novel molecules targeting these three distinct immune regulatory pathways; αTIGIT, to block TIGIT:CD155, and bintrafusp alfa, a bifunctional fusion protein targeting both TGFβ signaling and PD-1/PD-L1 negative regulation.

In the MC38-CEA colon carcinoma model, we observed a significant reduction in tumor volumes in the cohort treated with αTIGIT + bintrafusp alfa in comparison to untreated animals, or cohorts that receive either molecule as monotherapy (Figure 2b), as well as an increase in median survival (Figure 2c). Antitumor responses elicited by the combination therapy were dependent on CD4^+^ and CD8^+^ T cells (Figure 3) and were also associated with the generation of tumor antigen-specific T cells (Figure 5f). Furthermore, αTIGIT + bintrafusp alfa treatment resulted in a 50% tumor-free survival, with the cured animals acquiring protection from tumor re-challenge (Figure 2 d and e). Overall, the data provide evidence that the combination treatment strategy can stimulate antitumor T cells and generate long-lived memory responses in the MC38-CEA tumor model.

TIGIT expression is upregulated on a multitude of human cancers, including HPV^+^ malignancies. Intriguingly, HPV^+^ and HPV^−^ HNSCC possess molecularly distinct landscapes and subsequently variability in clinical outcome.^42^ It has been reported that HPV^+^ tissues, in comparison to HPV^−^ or adjacent cancer-free tissues, have significantly higher expression of LAG3, PD-1, TIGIT, and TIM3.^42^ Utilizing the HPV16 E6 and E7 expressing cell line, TC1, we observed significant antitumor activity with αTIGIT + bintrafusp alfa combination therapy (Figure 6b), associated with the appreciable increase in CD45^+^ cells, CD8^+^ T cells, NK cells, M-MDSCs, and DCs (Figure 6c).

Several studies demonstrate that CD226 is required for the stimulatory effect of both TIGIT and PD-1/PD-L1 blockade.^14,37,43^ Furthermore, TIGIT and PD-1 receptors, through separate and distinct mechanisms, partly exert their inhibitory functions through the impairment of CD226 signaling, emphasizing the necessity for dual blockade.^44^ While we observed a two-fold decrease in bioavailable TIGIT in tumor infiltrating CD8^+^ T cells in the MC38-CEA model, we did not detect the same reduction in the TC1 model. We also did not observe any changes in CD226 expression on CD8^+^ T cells upon αTIGIT + bintrafusp alfa treatment in both models. Further investigation on the phosphorylation and activation status of CD226 will be required to elucidate the role of this molecule in the therapeutic effect of our combination therapy regimen.

The bifunctional fusion protein bintrafusp alfa has recently undergone three late-stage clinical failures. The clinical trial NCT03631706 studied a head-to-head comparison of bintrafusp alfa versus pembrolizumab monotherapy in patients with NSCLC, where the novel molecule failed to outperform the standard of care. Trial NCT03833661 investigated the benefit of bintrafusp alfa as monotherapy in locally advanced or metastatic biliary tract cancer (BTC) but was halted due to an objective response rate of just 10%. The third trial, NCT04066491, aimed to determine efficacy of bintrafusp alfa in combination with gemcitabine and cisplatin in locally advanced or metastatic BTC and was discontinued upon determination that the study was unlikely to improve overall survival. However, preliminary clinical trial data from our group demonstrate that bintrafusp alfa has significant activity as a monotherapy in patients with HPV-associated malignances, resulting in a median overall survival (OS) of 21.3 months in comparison to OS ≤ 12 months following anti-PD-1/PD-L1 therapy^9^ (NCT02517398 and NCT03427411). Herein, we report similar results of efficacy with our αTIGIT + bintrafusp alfa combination therapy and purport this as a rationale progression in treatment of patients with HPV-associated malignancies.

Tumors exist on a rheostat of immune infiltrated (“hot” tumors) to immune excluded (“cold” tumors).^45^ Important caveats when interrogating ICB are the requirement of preexisting cells in the TME or the ability of effector cells to access the TME following ICB. Herein, we reported that Responders to combination treatment with αTIGIT + bintrafusp alfa have significant immune cell infiltrate of CD45^+^ cells, Tregs, CD8^+^ T cells, NK cells, M-MDSCs, PMN-MDSCs, macrophages, and DCs into the MC38-CEA TME (Figure 4). This is further supported by transcriptomic data showing an increase in expression of genes involved in immune cell adhesion/migration (CD28, Dsc3, Icos, Itga2, Pdcd1, Sele) and genes involved in chemokine and cytokine signaling (CCL20, CCL21α, CCR9, Csf2, CXCL3, IL-10, IL-23 R, IL-12Rβ2) when comparing Responders to untreated cohorts (Figure 7 b and d). Furthermore, we reported significant upregulation in genes involved in immune cell adhesion/migration (Dsc3, Olr1) and genes involved in chemokine and cytokine signaling (CCL21α, IL-12Rβ2, Nod2) between Responders and Non-Responders (Figure 8 a and b; online supplemental Table 1). These data act in concert to support increased infiltration in cohorts that responded to combination therapy of αTIGIT + bintrafusp alfa (summarized in Figure 7 c and d).

Directing immune cells into the TME is an obstacle that must be overcome to develop meaningful responses to ICB, but it is not the only one. Effectiveness of immunomodulatory agents also depends on effector cell ability to avoid exhaustion and perform their intended effector function. Dual expression of PD-1 and LAG3 on T cells has traditionally been considered a marker of a hyper-exhausted phenotype; however, emerging evidence indicates these cells are, in fact, tumor-specific, have increased cytotoxicity and proliferative capacity, and are more activated.^46^ We observed both increased expression levels of LAG3 (Figure 4f), PD-1 (Figure 4g) and an increased frequency of PD-1^+^LAG3^+^CD8^+^ T cells (Figure 4e) in Responders in comparison to other treatment groups. A two-fold increase in PD-1^+^LAG3^+^CD8^+^ T cells was also observed in the TC1 model with the combination treatment (Figure 6e). Additionally, we reported an increase in tumor antigen-specific CD8^+^ T cells from the αTIGIT + bintrafusp alfa Responders cohort in comparison to untreated control animals on day 17 and day 24 post-tumor inoculation (Figure 5 f and g, left panels). The frequency of tumor-specific CD8^+^ T cells was inversely correlated with tumor volume, with the smallest tumors generating the most robust tumor-reactivity (Figure 5 f and g, right panels). Additionally, a retrospective analysis in a lung cancer cohort revealed greater overall survival in patients who had CD8^+^ TILs that expressed high levels of PD-1, LAG3, and TIM-3.^46^ These results verify the increase of CD8^+^ T cells that are co-expressing traditional exhaustion markers are not terminally exhausted, but rather still capable of secreting IFNγ in response to tumor antigen, as well as having increased cytotoxicity as evidenced by elevated Prf1 (Figure 7b).

To further support our functional findings, interrogation of the TME revealed significant increases in genes responsible for immune activation of Responders in comparison to untreated controls (Apol6, CD28, FasL, Fcer1a, Icos, IL-12Rβ2, Irf4, Pdcd1, Prf1, TNFRSF4, TNFSF18) (Figure 7b-d), as well as increases in immune activation in Responders in comparison to Non-Responders (Fcer1a, IL-12Rβ2, Prf1, TNFRSF4, and TNFRSF18) (Figures 6 c and d, 7a and b). Additionally, several genes encoding coinhibitory receptors, such as B7-H4 and GITRL, are significantly downregulated in Responders in comparison to Non-Responders (Figure 8a). These data indicate that although we have a global increase of several classically defined positive and negative immune cell subsets, the TME of Responders versus Non-Responders and untreated cohorts remains immunostimulatory, with greater antigen-specificity of cytotoxic T cells (Figures 4 c-g, 5f and g, 6a-d, 7a and b).

PD-1/PD-L1, TIGIT, and TGFβ all contribute to the suppressive function of Tregs and disruption of these pathways has been employed to modulate Treg activity.^47^ While the αTIGIT + bintrafusp alfa treatment neither decreased Treg populations nor improved CD8:Treg ratio in the MC38-CEA and TC1 tumor models, it decreased the TIGIT:CD226 ratio on tumor infiltrating Tregs in both models. Ligation of TIGIT with CD155 promotes the suppressive functions of Tregs, while CD155:CD226 interactions impede Treg activity and stability.^33^ Moreover, upon interrogation of DEGs in Responders compared to Non-Responders, several DEGs are involved in mast cell degranulation (Cpa3, Tpsab1, etc.) (Figure 8a). Previous reports indicate mast cell degranulation in colorectal carcinomas are a result of the inability of Tregs to suppress a pro-inflammatory environment.^48^ Furthermore, in human colorectal cancer, tumor infiltrating Tregs lose their suppressive capacity and adopt Th17-like features.^49^ We, therefore, posit that Tregs found within the TME of our model are incapable of suppressing antitumor effector immune responses.

Cytokines have emerged as playing an important, albeit poorly understood, role in tumor progression.^50^ When we evaluated peripheral cytokine levels in MC38-CEA tumor-bearing mice 14 days after tumor instillation, we noted increased levels of IL-1β in animals receiving αTIGIT + bintrafusp alfa that responded to therapy (Figure 5a). IL-1β is pleiotropic in nature, possessing both pro- and antitumor activity. IL-1β favors commitment of T helper cells toward a proinflammatory Th17 phenotype.^51^ IL-1β is primarily produced by cells of the monocytic lineage, such as macrophages, which remains consistent with observed intratumoral increase in the myeloid compartment (Figure 4c). In addition, transcriptomic evidence reveals Nod2, which leads to increased secretion of IL-1β from macrophages resulting in a cascade of proinflammatory cytokines, is significantly elevated in αTIGIT + bintrafusp alfa Responders in comparison to Non-Responders (Figure 8). We observed a significant decrease in peripheral IL-2 in αTIGIT + bintrafusp alfa Responders in comparison to all other cohorts (Figure 5b). While several reports highlight the ability of IL-2 to activate effector cells, other IL-2 therapy studies reveal that this cytokine can promote Tregs in cancer patients.^52,53^ Interestingly, a study in patients with ovarian carcinoma showed that after IL-2 therapy cessation, Tregs populations in Responders significantly dropped when compared to Non-Responders.^53^ We posit that in the untreated mouse cohort, the high-affinity receptor expression bias allows Tregs to outcompete CD8^+^ T cells in the TME for access to IL-2, essentially shutting down CD8^+^ T cell effector function which was reversed by the combination therapy-induced decrease in IL-2.

In our studies, we observed a significant reduction in TGFβ in the αTIGIT + bintrafusp alfa Responders cohort in comparison with untreated animals, while Non-Responders failed to modulate TGFβ concentrations (Figure 5e). In addition, Reln, a protein involved in TGFβ-induced migration and metastasis of cancer cells, is found at significantly reduced levels in Responders in comparison to Non-Responders (Figure 8a).

We report for the first-time αTIGIT in combination with bintrafusp alfa results in prominent antitumor activity and increase in overall survival in both the MC38-CEA colon carcinoma and the TC1, HPV^+^ lung carcinoma models, which are dependent on CD4^+^ and CD8^+^ T cells. This combination treatment results in immune cell infiltration into the tumors, increased activation, and cytotoxicity of TIL. αTIGIT + bintrafusp alfa Responders display a more immune-activated landscape, in cytokine measurements, TIL, and transcriptomic profiles. These data represent potential indicators of response or resistance to therapy that could be monitored in patients enrolled in clinical trials.

## Supplementary Material

Supplemental Material

## Data Availability

Data will be made available upon reasonable request.
