# Supplementary material for: Immune targeting of three independent suppressive pathways (TIGIT, PD-L1, TGFβ) provides significant antitumor efficacy in immune checkpoint resistant models
Source: Oncoimmunology. 2022 Oct 1;11(1):2124666. doi: 10.1080/2162402X.2022.2124666 (PMC9542338; doi:10.1080/2162402X.2022.2124666)
Supplement: Supplemental Material [file KONI_A_2124666_SM1428.pdf]

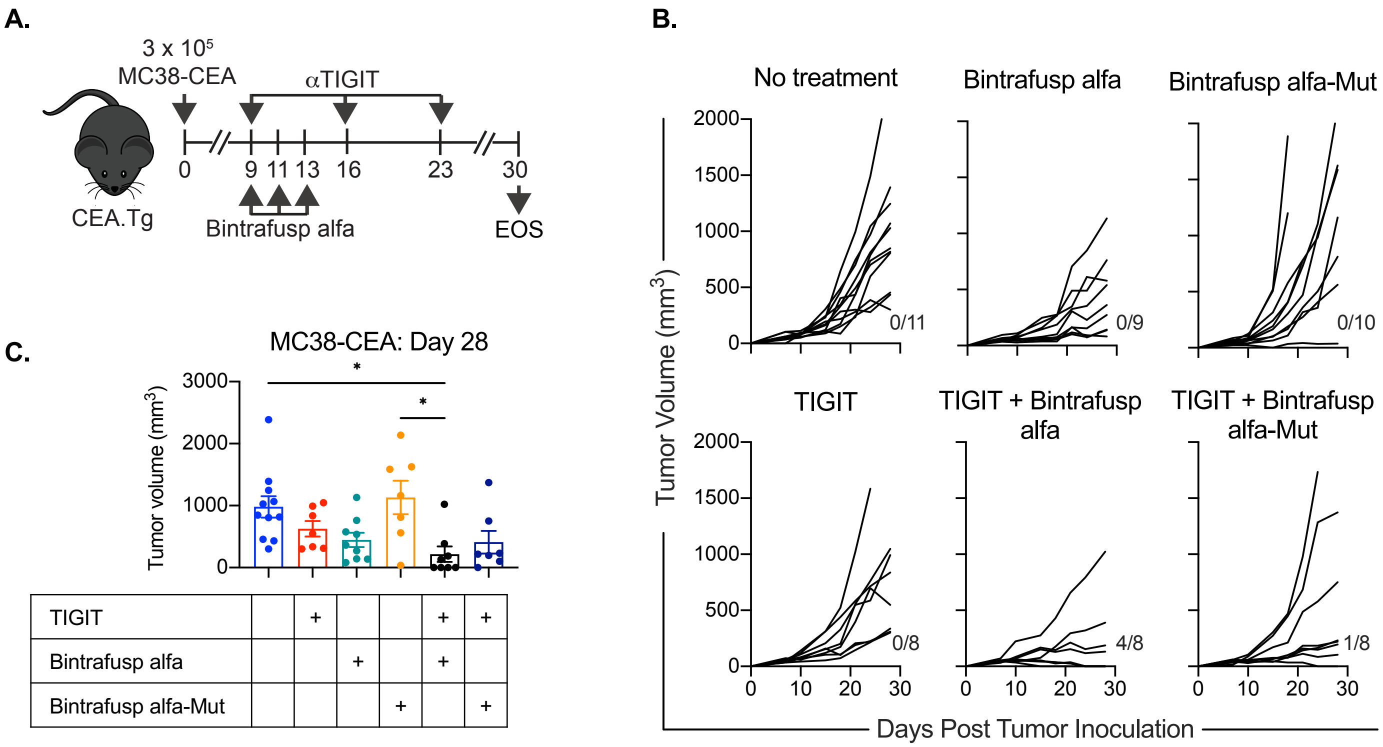


**Supplemental Figure 1. αTIGIT and bintrafusp alfa synergize to control MC38-CEA tumor growth and requires PD-L1 binding.** (A) Graphical representation of experimental design MC38-CEA tumor growth curves of CEA.Tg mice treated with bintrafusp alfa (n=9), a mutated version of bintrafusp alfa (designated bintrafusp alfa-Mut) that traps TGF-β but does not bind PD-L1 (n=10), αTIGIT (n=8), , αTIGIT + bintrafusp alfa (n=8), αTIGIT + bintrafusp alfa-Mut (n=8) or untreated controls (n=11). EOS = end of study. Numbers at the bottom right of tumor growth rate plots indicate tumor-free mice. (B) Tumor growth curves. (C) Day 28 average tumor volumes. *=p<0.05.

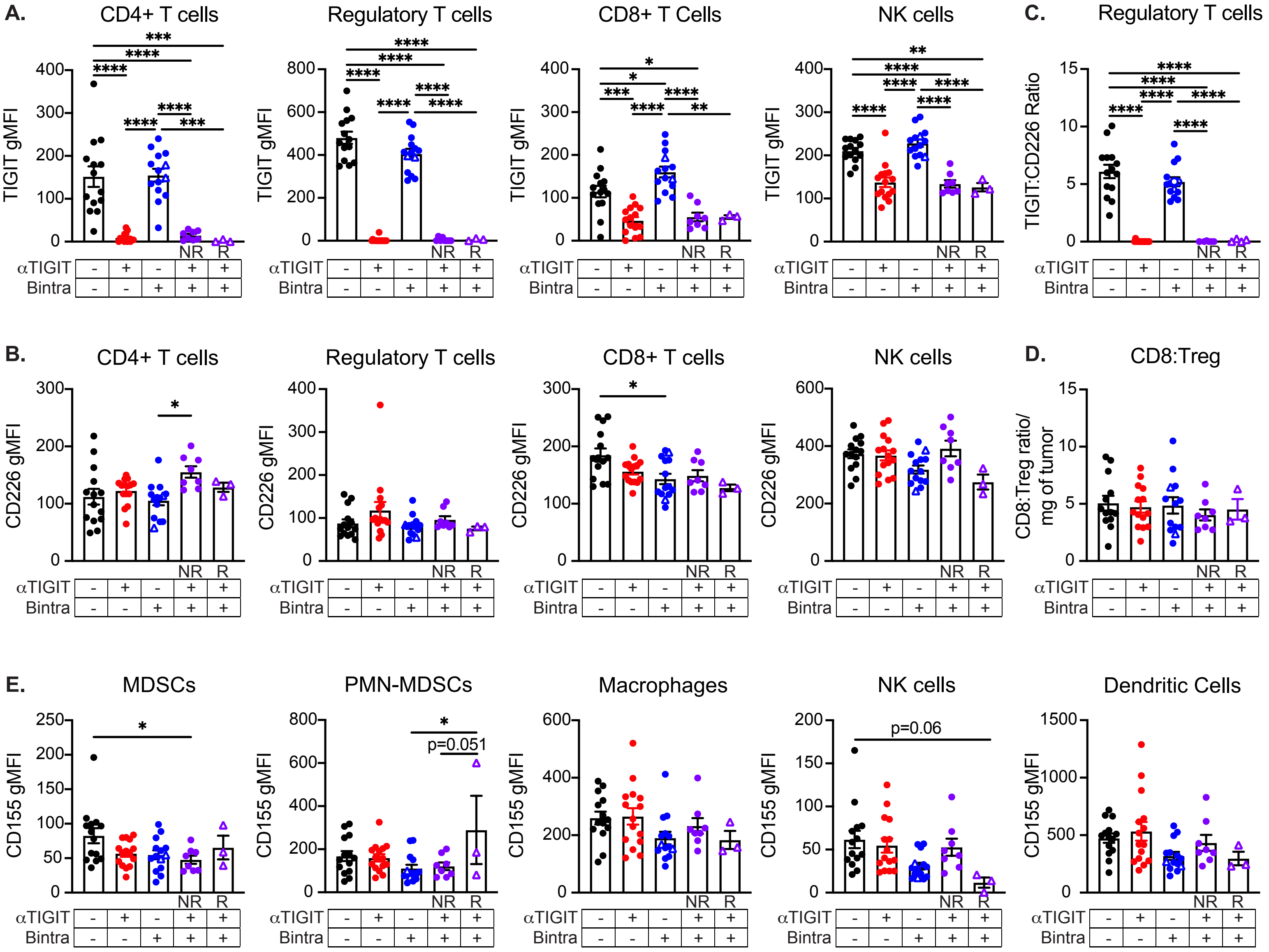


**Supplemental Figure 2. TIGIT, CD226 and CD155 expression levels on tumor infiltrating lymphocytes in the MC38-CEA model.** At day 24 post tumor inoculation, tumors from mice treated with αTIGIT (n=15), bintrafusp alfa (n=14), αTIGIT + bintrafusp alfa Non-Responders (n=8), αTIGIT + bintrafusp alfa Responders (n=3) and untreated animals (n=14) were excised and additional TIL analysis was performed (see figure 4A for graphical representation). (A) Quantification of TIGIT and (B) CD226 expression on CD4+, Treg, CD8+ and NK cells. (C) Quantification of TIGIT:CD226 ratio on regulatory T cells. (D) Quantification of CD8+ effector to Treg ratio. (E) Quantification of CD155 expression levels on monocytic myeloid derived suppressor cells (MDSCs), polymorphonuclear MDSCs (PMN-MDSCs), macrophages, NKs and DCs. NR = Non-Responders. R = Responders. gMFI = geometric mean fluorescent intensity. *=p<0.05, **=p<0.01, ***=p<0.005, ***=p<0.0001.

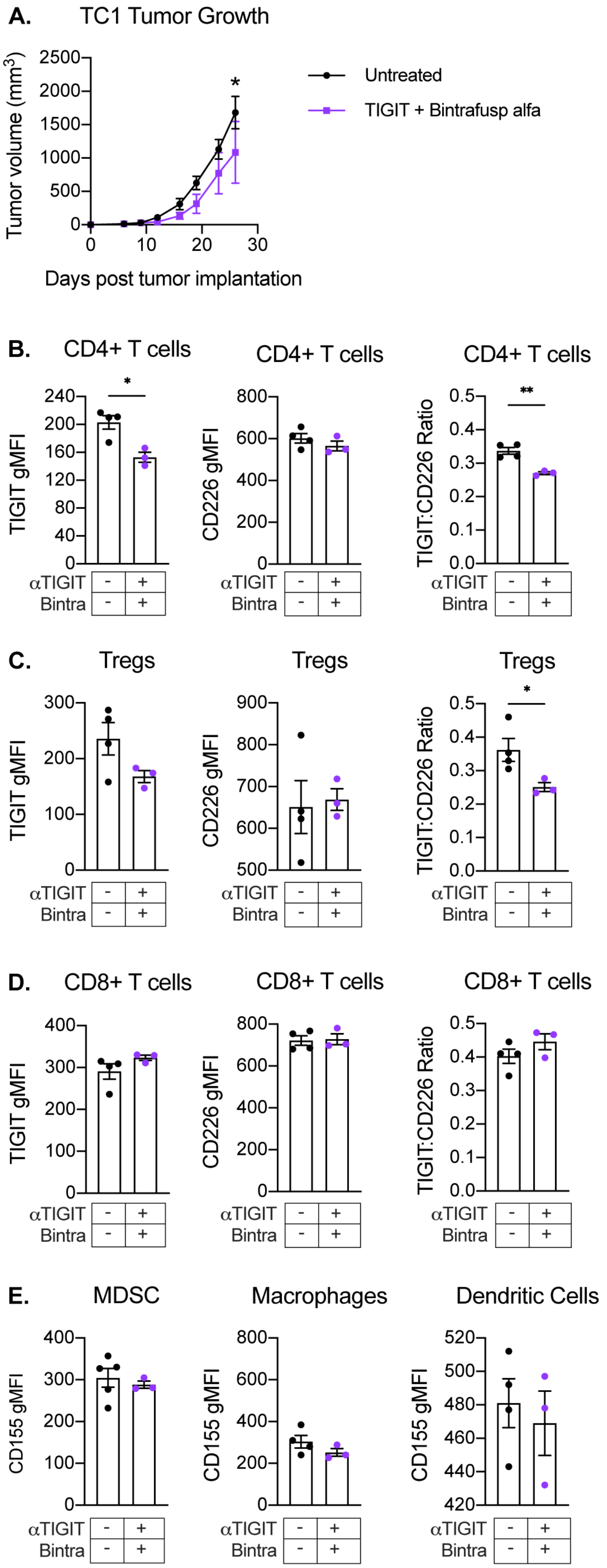


**Supplemental Figure 3. TIGIT, CD226 and CD155 expression levels on tumor infiltrating lymphocytes in the TC1 model.** (A) TC1 tumor growth curves of C57BL/6 mice that were treated with αTIGIT + bintrafusp alfa (purple line; *n*=3) or were left untreated (black line; *n*=4; see figure 6A for treatment schedule). On day 26 post tumor inoculation, tumors were excised and indicated immune cell subsets were interrogated via flow cytometry. Quantification of TIGIT expression, CD226 expression, and TIGIT:CD226 ratio on (B) CD4$^{+}$, (C) regulatory, and (D) CD8 T cells. (E) Quantification of CD155 expression levels on MDSCs, macrophages, dendritic cells. Tregs = regulatory T cells. MDSC = myeloid-derived suppressor cells. gMFI = geometric mean fluorescent intensity. *=p<0.05.

| Gene ID | Immune Cell Adhesion/ Migration | Immune Activation | Immune Regulation | Matrix Remodeling | Myeloid Compartment | Cytokine and Chemokine | Tumor Progression | Direction of Change |
|---|---|---|---|---|---|---|---|---|
| Ccl21a | | | | | | + | | ↑ |
| Cpa3 | | | | | + | | | ↑ |
| Dsc3 | + | | | + | | | | ↑ |
| Fcer1a | | + | | | + | | | ↑ |
| Foxp3 | | | + | | | | | ↑ |
| *Gbp2b* | | | | | | | | ↑ |
| Il12rb2 | | + | | | | + | | ↑ |
| Ms4a2 | | | | | + | | | ↑ |
| Nod2 | | | | | | + | | ↑ |
| Olr1 | + | | | | + | | | ↑ |
| Prf1 | | + | | | | | | ↑ |
| *Ren1* | | | | | | | | ↑ |
| Tnfrsf4 | | + | | | | | | ↑ |
| Tnfrsf18 | | + | | | | | | ↑ |
| Tpsab1 | | | | | + | | | ↑ |
| Ccl24 | | | | | | + | | ↓ |
| F2rl1 | | | | | | | + | ↓ |
| Ifna1 | | + | | | | | | ↓ |
| Il11ra1 | | | + | | | + | | ↓ |
| Lamb3 | + | | | + | + | | | ↓ |
| *Mgmt* | | | | | | | | ↓ |
| pcdhb11 | | | | | | | + | ↓ |
| Prr5 | | + | | | | | + | ↓ |
| Reln | | | | | | | + | ↓ |
| Robo4 | | | | | | | + | ↓ |
| S100a9 | | | | | + | | | ↓ |
| Siglecf | | | | | + | | | ↓ |
| Tnfsf18 | | + | | | | | | ↓ |
| Vtcn1 | | | + | | | | | ↓ |
| Wnt10a | | | | | | | + | ↓ |

**Supplemental Table 1. Differentially expressed genes in αTIGIT + bintrafusp alfa Responders versus αTIGIT + bintrafusp alfa Non-Responders affect the immune profile.** Seven distinct categories relating to the immune profile were created for differentially expressed genes as identified by the NanoString nCounter® PanCancer Pathways Panel. These categories were built based on data from NanoString and current literature.

| Gene ID | IFN | JAK-STAT | MAPK | NFkB | Notch | PI3K | TGFb | Wnt | Direction of Change |
|---|---|---|---|---|---|---|---|---|---|
| Gbp2b | + | | | | | | | | ↑ |
| Il12rb2 | | + | | | | | | | ↑ |
| Tnfrsf18 | | | | + | | | | | ↑ |
| Tnfrsf4 | | | | + | | | | | ↑ |
| Ifna1 | + | + | | | | + | | | ↓ |
| Il11ra1 | | + | | | | | | | ↓ |
| Lamb3 | | | | | | + | | | ↓ |
| Reln | | | | + | | + | | | ↓ |
| Tnfsf18 | | | | + | | | | | ↓ |
| Wnt10a | | | | | | | | + | ↓ |

**Supplemental Table 2. Differentially expressed genes in αTIGIT + bintrafusp alfa Responders versus αTIGIT + bintrafusp alfa Non-Responders affect the signaling profile.** Eight distinct categories relating to the signaling profile were created for differentially expressed genes as identified by the NanoString nCounter® PanCancer Pathways Panel. These categories were built based on data from NanoString and current literature.
